# A phase-II trial of dose-dense chemotherapy in patients with disseminated thymoma: report of a Japan Clinical Oncology Group trial (JCOG 9605)

**DOI:** 10.1038/sj.bjc.6605347

**Published:** 2009-10-06

**Authors:** H Kunitoh, T Tamura, T Shibata, K Nakagawa, K Takeda, Y Nishiwaki, Y Osaki, K Noda, A Yokoyama, N Saijo, JCOG Lung Cancer Study Group, Tokyo, Japan

**Affiliations:** 1 Department of Medical Oncology, National Cancer Center Hospital, 5-1-1 Tsukiji, Chuo-ku, Tokyo 104-0045, Japan; 2 JCOG Data Center, Center for Cancer Control and Information Services, National Cancer Center; 5-1-1 Tsukiji, Chuo-ku, Tokyo 104-0045, Japan; 3 Department of Medical Oncology, Kinki University School of Medicine, 377-2 Ohnohigashi, Osakasayama, Osaka 589-8511, Japan; 4 Department of Medical Oncology, Osaka City General Hospital, 2-13-22 Miyakojima-Hondori, Miyakojima-ku, Osaka 534-0021, Japan; 5 Department of Thoracic Oncology, National Cancer Center Hospital East, 6-5-1 Kashiwanohara, Kashiwashi, Chiba 277-8577, Japan; 6 Department of Internal Medicine, Asahikawa Medical College, 1-1-1 Higashinijou, Midorigaoka, Asahikawa, Hokkaido 078-8510, Japan; 7 Division of Thoracic Oncology, Kanagawa Cancer Center, 1-1-2 Nakao, Asahi-ku, Yokohama, Kanagawa 241-0815, Japan; 8 Department of Medical Oncology, Niigata Cancer Center, 2-15-3, Kawagishi-cho, Niigata-shi, Niigata 951-8566, Japan; 9 Department of Respiratory Medicine, Mitsui Memorial Hospital, 1 Kandaizumicho, Chiyoda-ku, Tokyo 101-8643, Japan; 10 National Cancer Center Hospital East, 6-5-1 Kashiwanoha, Kashiwashi, Chiba 277-8577, Japan

**Keywords:** thymoma, chemotherapy, dose-dense, platinum, anthracycline, granulocyte colony-stimulating factor

## Abstract

**Background:**

To evaluate the safety and efficacy of dose-dense weekly chemotherapy in the treatment of advanced thymoma.

**Methods:**

Subjects comprised patients with histologically documented chemotherapy-naïve thymoma with stage-IVa or IVb disease. Thymic carcinoma, carcinoid or lymphoma cases were excluded. Patients received 9 weeks of chemotherapy: cisplatin (25 mg m^−2^) on weeks 1–9; vincristine (1 mg m^−2^) on weeks 1, 2, 4, 6 and 8; and doxorubicin (40 mg m^−2^) and etoposide (80 mg m^−2^) on days 1–3 of weeks 1, 3, 5, 7 and 9. Chemotherapy courses were supported by granulocyte colony-stimulating factor. Post-protocol local therapy was allowed.

**Results:**

From July 1997 to March 2004, 30 patients were entered. Three were ineligible due to different histology. Chemotherapy-associated toxicity was mainly haematological and was well tolerated, with no deaths due to toxicity, and 87% of patients completed the planned 9-week regimen. Overall response rate was 59%, with 16 of the 27 eligible patients achieving partial response. Median progression-fee survival (PFS) was 0.79 years (95% confidence interval: 0.52–1.40 years), and PFS at 1 and 2 years was 37 and 15%, respectively. Overall survival rates at 2 and 5 years were 89 and 65%, respectively.

**Conclusion:**

In stage-IV thymoma patients, weekly dose-dense chemotherapy offers similar activity to conventional regimens.

Thymoma is a rare thoracic tumour, but remains one of the most common tumours originating in the mediastinum (Thomas *et al*, 1999; Giaccone, 2005; Girard *et al*, 2009). Clinical behaviour tends to be indolent, but dissemination into the pleural space eventually occurs and sometimes distant metastasis arise (Thomas *et al*, 1999). Thymoma is frequently associated with paraneoplastic syndromes such as myasthenia gravis or pure red cell aplasia (Thomas *et al*, 1999; Giaccone, 2005). No International Union Against Cancer (UICC) TNM classification is available, and the Masaoka classification has been widely used for clinical staging (Masaoka *et al*, 1981; Girard *et al*, 2009).

The majority of thymomas are discovered at a limited stage, representing Masaoka stage-I or II, and surgical resection is the treatment of choice for such cases (Thomas *et al*, 1999; Giaccone, 2005; Girard *et al*, 2009). Even when the tumour invades neighbouring organs, as stage-III disease, surgical resection with postoperative radiotherapy is the preferred treatment when complete resection can be achieved (Curran *et al*, 1988; Urgesi *et al*, 1990; Ogawa *et al*, 2002; Strobel *et al*, 2004).

Systemic chemotherapy is usually used for stage-IVa (with pleural or pericardial dissemination) or stage-IVb disease (with lymphogenous or haematogenous metastases), but optimal management is less well established (Thomas *et al*, 1999; Girard *et al*, 2009). Several reports have described favourable outcomes in limited numbers of patients with stage-IVa disease treated using multimodal treatment including surgery (Kim *et al*, 2004; Yokoi *et al*, 2007).

Conversely, thymomas are generally reported to be chemotherapy-sensitive tumours, with response rates of 50–70% to combination chemotherapy (Fornasiero *et al*, 1990; Loehrer *et al*, 1994, 1997, 2001; Giaccone *et al*, 1996; Berruti *et al*, 1999; Kim *et al*, 2004; Lucchi *et al*, 2006; Yokoi *et al*, 2007). Active agents include cisplatin (CDDP), vincristine (VCR), doxorubicin (ADM), etoposide (ETP), cyclophosphamide (CPM) and ifosfamide (IFX). Recent reports have shown marginal activity of pemetrexed (Loehrer *et al*, 2006) and combined carboplatin and paclitaxel (Lemma *et al*, 2008).

Dose-dense chemotherapy with the CODE combination (CDDP–VCR–ADM–ETP) and addition of granulocyte colony-stimulating factor (G-CSF) can be safely administered to patients with advanced lung cancer (Murray *et al*, 1991; Fukuoka *et al*, 1997). Theoretically, this approach might be suitable for chemosensitive tumours such as small-cell lung cancer and thymoma (Goldie and Coldman, 1983, 1984; Levin and Hryniuk, 1987; Murray, 1987). Because some pilot data in Japan suggested that administration of 12 weeks of the CODE chemotherapy was barely feasible, subsequent Japanese trials used a modified schedule, which was shortened to 9 weeks (Fukuoka *et al*, 1997; Furuse *et al*, 1998).

In 1996, the Japan Clinical Oncology Group (JCOG) initiated two clinical trials for advanced thymoma: one aimed at evaluating the safety and efficacy of the CODE regimen in stage IV, disseminated thymoma (JCOG 9605), and the other aimed at evaluating the safety and efficacy of CODE combination chemotherapy followed by surgical resection and postoperative radiotherapy in initially unresectable stage-III thymoma (JCOG 9606). The primary endpoint in each study was progression-free survival (PFS). The results of JCOG 9605 are reported herein.

## Patients and methods

### Eligibility criteria

Patients with chemotherapy-naive, histologically documented thymoma at Masaoka stage IVa or IVb were eligible for entry into the study. Thymoma must have been confirmed histologically and thymic tumours with other histology, such as thymic carcinoma, carcinoid or lymphoma, were excluded. Each patient was required to fulfil the following criteria: age, 15–70 years; Eastern Cooperative Oncology Group (ECOG) performance status (PS), 0–2; adequate organ function, that is, leukocyte count ⩾4000 *μ*l^−1^, platelet count ⩾10^5^ *μ*l^−1^, hemoglobin ⩾10.0 g dl^−1^, serum creatinine <1.5 mg dl^−1^, creatinine clearance ⩾60 ml min^−1^, serum bilirubin <1.5 mg dl^−1^, serum alanine transaminase and aspartate transaminase levels less than double the upper limit of the institutional normal range; and PaO_2_ ⩾70 mm Hg. Exclusion criteria included uncontrolled heart disease, uncontrolled diabetes or hypertension, pulmonary fibrosis or active pneumonitis as evidenced on chest radiography, infections necessitating systemic use of antibiotics, disease necessitating emergency radiotherapy such as superior vena cava obstruction syndrome, active concomitant malignancy and women who were pregnant or lactating. Also excluded were those patients with grave complications of thymoma, such as pure red cell aplasia or hypogammaglobulinemia. Myasthenia gravis was allowed and these patients were not excluded *per se*.

Patient eligibility was confirmed by the JCOG Data Center before patient registration. This study protocol was approved by the institutional review board at each participating centre and written informed consent was obtained from all patients prior to enrolment.

### Treatment Plan

#### Chemotherapy

Patients received the 9-week CODE combination chemotherapy as described below. Each chemotherapeutic agent was administered intravenously.

Week 1: CDDP 25 mg m^−2^ on day 1 with antiemetics and ample hydration; VCR (1 mg m^−2^) on day 1; ADM (40 mg m^−2^) on day 1 and ETP (80 mg m^−2^) on days 1–3.

Weeks 2, 4, 6 and 8: CDDP (25 mg m^−2^) on day 1 with antiemetics and ample hydration and VCR (1 mg m^−2^) on day 1.

Weeks 3, 5, 7 and 9: CDDP (25 mg m^−2^) on day 1 with antiemetics and ample hydration, ADM (40 mg m^−2^) on day 1 and ETP (80 mg m^−2^) on days 1–3.

Each week, G-CSF (filgrastim (50 *μ*g m^−2^ day^−1^) or lenograstim (2 *μ*g kg^−1^ day^−1^)) was administered by subcutaneous injection, except on days when chemotherapy was administered or when leukocyte count was ⩾10 000 *μ*l^−1^. Corticosteroid was used only as part of the antiemetic regimen, and the specific drug and dosage were not regulated by the protocol.

Dose and schedule modifications were performed as follows: when leukocyte count decreased to <2,000 *μ*l^−1^ or platelet count decreased to <50 000 *μ*l^−1^, chemotherapy was delayed by 1 week. If PS decreased to 3–4 or temperature reached ⩾38.0°C, therapy was likewise delayed for 1 week. No dose modification of chemotherapy drugs was adopted for toxicity.

### Post-protocol therapy

Surgery or radiotherapy was allowed after the completion of chemotherapy, at the discretion of the attending physician, even in the absence of apparent tumour regrowth. Conversely, additional chemotherapy without evidence of disease progression was not allowed.

Post-treatment after disease progression was not limited by the study protocol.

### Patient evaluation and follow-up

Before enrolment into the study, each patient underwent complete medical history taking and physical examination (including neurological check-up for signs of myasthenia gravis), determination of blood cell counts, serum biochemistry testing, arterial blood gas analysis, pulmonary function testing, electrocardiography, chest radiography, computed tomography (CT) of the chest, CT or ultrasonography of the upper abdomen, whole-brain CT or magnetic resonance imaging (MRI) and an isotope bone scan. Blood-cell counts, serum biochemistry testing and chest radiography were performed weekly during each course of chemotherapy.

The toxicity of chemotherapy was evaluated according to the JCOG Toxicity Criteria (Tobinai *et al*, 1993), modified from version 1 of the National Cancer Institute Common Toxicity Criteria (NCI-CTC). Tumour responses were assessed radiographically according to the standard, two-dimensional WHO criteria (Miller *et al*, 1981), and were classified as complete response (CR), partial response (PR), no change (NC), progressive disease (PD) or non-evaluable (NE). After completion of the protocol therapy, patients were followed up with periodic re-evaluation, including chest CT every 6 months for the first 2 years and annually thereafter.

### Central review

Radiographic reviews for the eligibility of enrolled patients and clinical responses were performed at the time of the study group meeting, held every 3–4 months. The study coordinator (H Kunitoh) and a few selected investigators from the group reviewed the radiographic films. The clinical response data presented below were all confirmed by this central review. Reviews of pathological specimens were not performed, because of insufficient logistics of the study group at the time of the study activation in 1997.

### Endpoints and statistical considerations

The primary endpoint in each study was PFS. Due the rarity of the tumour and the accrual reported in US trials, which required 10 years to register 26 patients with locally advanced (stage-III) disease (Loehrer *et al*, 1997) and 9 years for 31 patients with disseminated (stage-IV) disease (Loehrer *et al*, 1994), we presumed we would be capable of accruing 30 patients in the target accrual period of 4 years. The sample size was, therefore, not determined based on statistical calculations. The expected PFS for the JCOG 9605 study was 2 years, which would give a 95% confidence interval of 1.3–3.0 years with 30 cases.

The initial study design thus envisioned enrolment of 30 fully eligible cases over 3 years for the study, with a follow-up period of 2 years.

Secondary endpoints included toxicity and safety, objective tumour response to chemotherapy, pattern of relapse, and overall survival (OS).

Progression-free survival and OS were calculated from the date of enrolment and estimated using the Kaplan–Meier method. Progression-free survival was censored at the last date verifiable as progression-free, and OS was censored as of the date of last follow-up. During the accrual period, an interim analysis for futility was planned after half of the patients had been registered and followed for ⩾3 months. All analyses were performed using SAS software version 8.2/9.1 (SAS Institute, Cary, NC, USA).

## Results

### Patient characteristics

A total of 30 patients from seven institutions were enrolled from July 1997 to March 2004. Three patients were later found ineligible due to wrong histology, with two cases of thymic carcinoma and one case of carcinoid. These mistakes occurred due to technical problems in the patient registry. Since the ineligible cases did receive the protocol therapy, all 30 patients were analysed for characteristics and toxicity. Twenty-seven eligible patients were analysed for clinical response and survival (PFS and OS). Patient characteristics are shown in Table 1.

### Chemotherapy delivery and toxicity

Nine weeks of chemotherapy were performed for 26 of the original 30 patients (87%). The other four patients included one patient receiving 7 weeks, two receiving 6 weeks and one receiving 3 weeks of therapy. Median duration of chemotherapy for the 26 patients who underwent the planned nine cycles was 10 weeks (range, 9–12 weeks).

Table 2 summarises the major toxicities of chemotherapy, which were mainly haematological. Although 70% of patients experienced grade-IV neutropenia, this was generally transient and rarely complicated by infection/fever. Overall, toxicities were well tolerated and no deaths due to toxicity occurred.

### Other and late complications

Four patients showed thymoma-related complications. One patient suffered from myasthenia gravis crisis occurring during chemotherapy, but subsequently recovered. Another patient showed newly diagnosed myasthenia gravis 2.5 years after completion of the protocol therapy, and thymectomy and resection of the residual tumour were performed. Two other cases had pure red cell aplasia occurring later in the clinical course with disease progression of the thymomas.

### Clinical response to chemotherapy

Clinical responses of the 27 eligible patients to chemotherapy were judged radiologically and confirmed by central review. Responses were as follows: CR, 0 patients; PR, 16 patients; NC, 10 patients and PD, 1 patient. Overall response rate was 59% (95% confidence interval, 39–78%).

### Post-protocol therapy

Post-protocol local therapy was administered to 18 of the 27 eligible patients (67%). Eight patients (all with stage-IVa disease) underwent surgical resection and 13 patients (nine with stage-IVa disease and four with stage-IVb disease) received thoracic radiotherapy, with three patients receiving both. Whether patients received local therapy after disease progression was not recorded on case report forms.

After disease progression, 16 of the 27 patients (59%) received additional chemotherapy. Post-protocol chemotherapy included platinum re-challenge, irinotecan, taxanes and investigational agents. Clinical response data to those therapies are not available.

### PFS and OS

**Figure 1:**
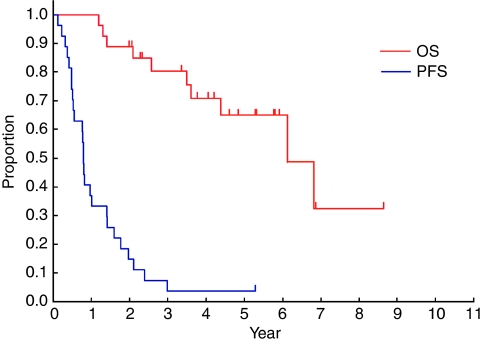
Progression-free survival and OS of the 27 eligible patients.

Survival data were finally updated in March 2006, 2 years after accrual of the last patient. Figure 1 shows PFS and OS curves of the 27 eligible patients. Median PFS was 0.79 years (95% confidence interval, 0.52–1.40 years) and PFS at 1 and 2 years was 37 and 15%, respectively. Median OS was 6.1 years and OS at 2 and 5 years was 89 and 65%, respectively.

**Figure 2:**
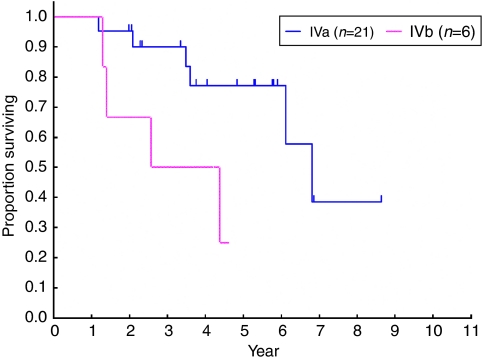
Overall survival according to Masaoka stage (stage IVa *vs* IVb).

**Figure 3:**
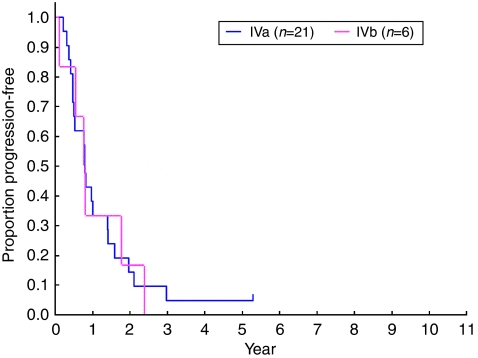
Progression-free survival according to Masaoka stage (stage IVa *vs* IVb).

Overall survival was longer for stage-IVa patients than for stage-IVb patients (Figure 2, median, 6.8 years and 3.5 years, respectively), but PFS was similar (Figure 3, median, 0.79 years for IVa patients and 0.78 years for IVb patients).

### Pattern of relapse

As of the data cut-off, 26 of the 27 eligible patients had experienced tumour relapse. Sites of initial relapse comprised the primary site only in seven cases (27%), pleural or pericardial dissemination in seven cases (27%) and primary site and pleural/pericardial dissemination in nine cases (35%). Thus, 23 of the 26 patients with relapse initially showed regrowth of the primary and/or pleural or pericardial dissemination, with only three patients (12%) showing initial relapse at distant organs.

## Discussion

Few prospective trials of chemotherapy have been described for patients with advanced thymoma. Most prior studies have combined stage-III, localised disease and stage-IV, disseminated disease (Table 3). In addition, most have also included both thymoma and thymic carcinoma histology.

We have reported results for patients with stage-IV disease, for which systemic therapy should be the first choice. Among previous studies, only those from the ECOG separately reported results for stage-III and stage-IV patients (Loehrer *et al*, 1994, 1997). The ECOG took 9 years to accrue 31 patients with stage-IV disease, including patients with thymic carcinoma (Loehrer *et al*, 1994). We prospectively accrued patients with thymoma only and excluded thymic carcinoma, as thymoma and thymic carcinoma clearly differ in clinical presentation and prognosis, and trials involving these pathologies should, thus, be reported separately (Eng *et al*, 2004; Giaccone, 2005; Lemma *et al*, 2008).

Trials of systemic chemotherapy for thymoma have reported response rates of 50–90%, so this tumour is generally considered sensitive to chemotherapy (Thomas *et al*, 1999). Dose-dense chemotherapy such as the CODE four-drug combination has been argued to be theoretically suitable for the treatment of such chemosensitive tumours (Murray, 1987).

Although our results showed that dose-dense CODE chemotherapy could be safely administered to thymoma patients, efficacy was not remarkable. The overall response rate was about 60%, no different from prior reports employing conventional-dose chemotherapy (Table 3). Progression-free survival was 9 months, falling far short of the expected 2 years. Although OS studies compared favourably with the corresponding ECOG trial (Loehrer *et al*, 1994), attempting to reach a valid conclusion would be difficult due to the small sample sizes. In addition, OS could be greatly affected by post-study local therapy especially in patients with stage-IVa disease, as combined therapy trial including stage-IVa patients suggested (Kim *et al*, 2004). In fact, this might be one reason why OS of stage-IVa patients was much longer than that of stage-IVb patients, whereas PFS was similar.

It could be argued that shortened CODE chemotherapy, used in Japan due to feasibility problem, led to inadequate results due to insufficient total dosages of chemotherapy drugs. However, another intensive chemotherapy, ETP–IFX–CDDP (VIP) supported by G-CSF, has also reported disappointingly low response rates and no better survival (Loehrer *et al*, 2001). Hanna *et al* (2001) reported five patients with prior chemotherapy treated with high-dose chemotherapy and stem cell support, but concluded that no superiority to conventional therapy was evident. Taken together with our results, intensification of chemotherapy does not appear sufficiently promising for treating advanced thymoma.

Many prior chemotherapy studies have included platinum and anthracyclines in their regimens. Non-anthracycline approaches contained regimens such as VIP (Loehrer *et al*, 2001), ETP–CDDP (Giaccone *et al*, 1996) and paclitaxel–carboplatin (Lemma *et al*, 2008) tended to yield lower response rates of 32–56% as compared with regimens including anthracycline (Table 3). It might, thus, be suggested that both anthracycline and platinum should, thus, be included in thymoma chemotherapy, at least in current clinical practice.

Favourable results have recently been reported with multimodality therapy, including surgical resection of stage-IVa disease (Kim *et al*, 2004; Yokoi *et al*, 2007). In fact, about two-thirds of eligible patients in our trial received local therapy after chemotherapy, including surgery in eight patients. This could have affected the outcome of the patients, as discussed above. However, small sample size and patient selection preclude reaching any definitive conclusion. When and what local therapy, if any, would benefit patients with disseminated thymoma, remains yet to be established. Further studies are warranted.

The present study shows several additional limitations. One is that we did not perform a central review of histology, and, thus, could not provide WHO classifications of histology (Okumura *et al*, 2002; Travis *et al*, 2004). This makes comparisons with results from other reports difficult. Central pathology review and preferably tissue collection would be very important in future trials.

In addition, due to the shorter-than-expected PFS, the planned CT scan interval of every 6 months might not have accurately evaluated PFS (Freidlin *et al*, 2007). Future trials might require more frequent scans.

In conclusion, we have reported that weekly dose-dense chemotherapy can be safely administered to patients with thymoma. However, efficacy seems similar to that in patients treated with conventional doses. More research on optimal systemic therapy and the role of local modalities would appear to be necessary.

## Figures and Tables

**Table 1:** Patient characteristics

**Item**	
*Sex*	
Male/female	16/14
	
*Age (years)*	
Median/range	47.5/29–69
	
*ECOG performance status*	
PS0/PS1/PS2	11/18/1
	
*Masaoka stage*	
IVa/IVb	22/8
	
*Smoking history*	
No	9
Yes (median pack–years)	21 (22)
	
*Myasthenia gravis*	
No/yes	28/2
	
*Histology: thymoma and eligible*	27
Lymphocyte predominance	12
Mixed cell	9
Epithelioid cell	4
Clear cell	1
Spindle cell	0
Unclassified	1
	
*Histology: not thymoma (ineligible)*	3
Carcinoma	2
Carcinoid	1
Lymphoma	0
	
*Prior therapy*	
None	26
Surgery	2
Surgery and radiation	2

Abbreviations: ECOG=Eastern Cooperative Oncology Group; PS=performance status.

**Table 2:** Toxicity of chemotherapy (*n*=30)

**Toxicity**	**Grades 1/2**	**Grade 3**	**Grade 4**	**%Grade 3/4**
Leukopenia	3/6	12	8	67
Neutropenia	3/1	5	21	87
Anemia	0/5	25	ND	83
Thrombocytopenia	4/6	5	3	27
ALT	9/0	0	0	0
Creatinine	2/1	0	0	0
PaO_2_	9/2	0	0	0
Emesis	13/11	2	ND	7
Diarrhoea	4/2	0	0	0
Stomatitis	4/3	0	0	0
Constipation	3/4	2	0	7
Neuropathy	11/2	0	ND	0
Infection	3/4	3	0	10

Abbreviations: ALT=alanine transaminase; ND=not defined (the JCOG toxicity criteria did not define grade IV in these toxicities).

**Table 3:** Reports of combination chemotherapy for thymoma

**Regimen**	**Stage**	**Patients** a	**ORR**	**Reference**
*Anthracycline-containing regimens*				
ADOC (S)	III/IV	32	91%	Fornasiero *et al* (1990)
PAC (G)	IV	30	50%	Loehrer *et al* (1994)
PAC (G)	III	23	70%	Loehrer *et al* (1997)
ADOC (S)	III/IV	16	81%	Berruti *et al* (1999)
PAC (G)	III/IV	22	77%	Kim *et al* (2004)
PAE (S)	III/IV	30	73%	Lucchi *et al* (2006)
CAMP (S)	III/IV	14	93%	Yokoi *et al* (2007)
CODE (G)	IV	27	59%	Current study
				
*Non-anthracycline-containing regimens*				
PE (G)	III/IV	16	56%	Giaccone *et al* (1996)
VIP (G)	III/IV	20	35%	Loehrer *et al* (1997)
CP (G)	III/IV	23	35%	Lemma *et al* (2008)

Abbreviations: ADOC=doxorubicin, cisplatin, vincristine, cyclophosphamide; CAMP=cisplatin, doxorubicin, methylpredonisolone; CODE=cisplatin, vincristine, doxorubicin, etoposide; CP=carboplatin, paclitaxel; G=prospective multicenter group trial; ORR=overall response rate; PAC=cisplatin, doxorubicin, cyclophosphamide; PAE=cisplatin, epidoxorubicin, etoposide; PE=cisplatin, etoposide; S=single-center experience; VIP=etoposide, ifosfamide, cisplatin.

a Number of assessable patients.

## Appendix 1

### Study participants

The following institutions and investigators participated in the trial:

Asahikawa Medical College (Yoshinobu Osaki), National Cancer Center Hospital East (Yutaka Nishiwaki, Kaoru Kubota, Nagahiro Saijo), National Cancer Center Hospital (Tomohide Tamura, Noboru Yamamoto, Hideo Kunitoh), Kanagawa Cancer Center (Kazumasa Noda, Fumihiro Oshita), Niigata Cancer Center Hospital (Akira Yokoyama, Yuko Tsukada), Kinki University Hospital (Kazuhiko Nakagawa, Isamu Okamoto) and Osaka City General Hospital (Koji Takeda, Haruko Daga).

## Appendix 2

### Acknowledgements

We thank Ms Mieko Imai for data management at the JCOG Data Center.

## References

[bib1] Berruti A, Borasio P, Gerbino A, Gorzegno G, Moschini T, Tampellini M, Ardissone F, Brizzi MP, Dolcetti A, Dogliotti L (1999) Primary chemotherapy with adriamycin, cisplatin, vincristine and cyclophosphamide in locally advanced thymomas: a single institution experience. Br J Cancer 81: 841–845 10555755 10.1038/sj.bjc.6690773 PMC2374302

[bib2] Curran Jr WJ, Kornstein MJ, Brooks JJ, Turrisi 3rd AT (1988) Invasive thymoma: the role of mediastinal irradiation following complete or incomplete surgical resection. J Clin Oncol 6: 1722–1727 3183702 10.1200/JCO.1988.6.11.1722

[bib3] Eng TY, Fuller CD, Jagirdar J, Bains Y, Thomas Jr CR (2004) Thymic carcinoma: state of the art review. Int J Radiat Oncol Biol Phys 59: 654–664 15183468 10.1016/j.ijrobp.2003.11.021

[bib4] Fornasiero A, Daniele O, Ghiotto C, Sartori F, Rea F, Piazza M, Fiore-Donati L, Morandi P, Aversa SM, Paccagnella A, Pappagallo GL, Fiorentino MV (1990) Chemotherapy of invasive thymoma. J Clin Oncol 8: 1419–1423 2380761 10.1200/JCO.1990.8.8.1419

[bib5] Freidlin B, Korn EL, Hunsberger S, Gray R, Saxman S, Zujewski JA (2007) Proposal for the use of progression-free survival in unblinded randomized trials. J Clin Oncol 25: 2122–2126 17513819 10.1200/JCO.2006.09.6198

[bib6] Fukuoka M, Masuda N, Negoro S, Matsui K, Yana T, Kudoh S, Kusunoki Y, Takada M, Kawahara M, Ogawara M, Kodama N, Kubota K, Furuse K (1997) CODE chemotherapy with and without granulocyte colony-stimulating factor in small-cell lung cancer. Br J Cancer 75: 306–309 9010043 10.1038/bjc.1997.50 PMC2063260

[bib7] Furuse K, Fukuoka M, Nishiwaki Y, Kurita Y, Watanabe K, Noda K, Ariyoshi Y, Tamura T, Saijo N (1998) Phase III study of intensive weekly chemotherapy with recombinant human granulocyte colony-stimulating factor versus standard chemotherapy in extensive-disease small-cell lung cancer. J Clin Oncol 16: 2126–2132 9626212 10.1200/JCO.1998.16.6.2126

[bib8] Giaccone G (2005) Treatment of malignant thymoma. Curr Opin Oncol 17: 140–146 15725919 10.1097/01.cco.0000152628.43867.8e

[bib9] Giaccone G, Ardizzoni A, Kirkpatrick A, Clerico M, Sahmoud T, van Zandwijk N (1996) Cisplatin and etoposide combination chemotherapy for locally advanced or metastatic thymoma. A phase II study of the European Organization for Research and Treatment of Cancer Lung Cancer Cooperative Group. J Clin Oncol 14: 814–820 8622029 10.1200/JCO.1996.14.3.814

[bib10] Girard N, Mornex F, Van Houtte P, Cordier JF, van Schil P (2009) Thymoma: a focus on current therapeutic management. J Thorac Oncol 4: 119–126 19096319 10.1097/JTO.0b013e31818e105c

[bib11] Goldie JH, Coldman AJ (1983) Quantitative model for multiple levels of drug resistance in clinical tumors. Cancer Treat Rep 67: 923–931 6627236

[bib12] Goldie JH, Coldman AJ (1984) The genetic origin of drug resistance in neoplasms: implications for systemic therapy. Cancer Res 44: 3643–3653 6744284

[bib13] Hanna N, Gharpure VS, Abonour R, Cornetta K, Loehrer Sr PJ (2001) High-dose carboplatin with etoposide in patients with recurrent thymoma: the Indiana University experience. Bone Marrow Transplant 28: 435–438 11593315 10.1038/sj.bmt.1703181

[bib14] Kim ES, Putnam JB, Komaki R, Walsh GL, Ro JY, Shin HJ, Truong M, Moon H, Swisher SG, Fossella FV, Khuri FR, Hong WK, Shin DM (2004) Phase II study of a multidisciplinary approach with induction chemotherapy, followed by surgical resection, radiation therapy, and consolidation chemotherapy for unresectable malignant thymomas: final report. Lung Cancer 44: 369–379 15140551 10.1016/j.lungcan.2003.12.010

[bib15] Lemma GL, Loehrer Sr PJ, Lee JW, Langer CJ, Tester WJ, Johnson DH (2008) A phase II study of carboplatin plus paclitaxel in advanced thymoma or thymic carcinoma: E1C99. J Clin Oncol 26(15S): abstract 8018 10.1200/JCO.2010.32.9607 PMC3107762 21502559

[bib16] Levin L, Hryniuk WM (1987) Dose intensity analysis of chemotherapy regimens in ovarian carcinoma. J Clin Oncol 5: 756–767 3572465 10.1200/JCO.1987.5.5.756

[bib17] Loehrer Sr PJ, Chen M, Kim K, Aisner SC, Einhorn LH, Livingston R, Johnson D (1997) Cisplatin, doxorubicin, and cyclophosphamide plus thoracic radiation therapy for limited-stage unresectable thymoma: an intergroup trial. J Clin Oncol 15: 3093–3099 9294472 10.1200/JCO.1997.15.9.3093

[bib18] Loehrer Sr PJ, Jiroutek M, Aisner S, Aisner J, Green M, Thomas Jr CR, Livingston R, Johnson DH (2001) Combined etoposide, ifosfamide, and cisplatin in the treatment of patients with advanced thymoma and thymic carcinoma: an intergroup trial. Cancer 91: 2010–2015 11391579

[bib19] Loehrer Sr PJ, Kim K, Aisner SC, Livingston R, Einhorn LH, Johnson D, Blum R (1994) Cisplatin plus doxorubicin plus cyclophosphamide in metastatic or recurrent thymoma: final results of an intergroup trial. The Eastern Cooperative Oncology Group, Southwest Oncology Group, and Southeastern Cancer Study Group. J Clin Oncol 12: 1164–1168 8201378 10.1200/JCO.1994.12.6.1164

[bib20] Loehrer Sr PJ, Yiannoutsos CT, Dropcho S, Burns M, Helft P, Chiorean EG, Nelson RP (2006) A phase II trial of pemetrexed in patients with recurrent thymoma or thymic carcinoma. J Clin Oncol 24(18S): abstract 7079

[bib21] Lucchi M, Melfi F, Dini P, Basolo F, Viti A, Givigliano F, Angeletti CA, Mussi A (2006) Neoadjuvant chemotherapy for stage III and IVA thymomas: a single-institution experience with a long follow-up. J Thorac Oncol 1: 308–313 17409875

[bib22] Masaoka A, Monden Y, Nakahara K, Tanioka T (1981) Follow-up study of thymomas with special reference to their clinical stages. Cancer 48: 2485–2492 7296496 10.1002/1097-0142(19811201)48:11<2485::aid-cncr2820481123>3.0.co;2-r

[bib23] Miller AB, Hoogstraten B, Staquet M, Winkler A (1981) Reporting results of cancer treatment. Cancer 47: 207–214 7459811 10.1002/1097-0142(19810101)47:1<207::aid-cncr2820470134>3.0.co;2-6

[bib24] Murray N (1987) The importance of dose and dose intensity in lung cancer chemotherapy. Semin Oncol 14: 20–28 2825357

[bib25] Murray N, Shah A, Osoba D, Page R, Karsai H, Grafton C, Goddard K, Fairey R, Voss N (1991) Intensive weekly chemotherapy for the treatment of extensive-stage small-cell lung cancer. J Clin Oncol 9: 1632–1638 1651995 10.1200/JCO.1991.9.9.1632

[bib26] Ogawa K, Uno T, Toita T, Onishi H, Yoshida H, Kakinohana Y, Adachi G, Itami J, Ito H, Murayama S (2002) Postoperative radiotherapy for patients with completely resected thymoma: a multi-institutional, retrospective review of 103 patients. Cancer 94: 1405–1413 11920495 10.1002/cncr.10373

[bib27] Okumura M, Ohta M, Tateyama H, Nakagawa K, Matsumura A, Maeda H, Tada H, Eimoto T, Matsuda H, Masaoka A (2002) The World Health Organization histologic classification system reflects the oncologic behavior of thymoma: a clinical study of 273 patients. Cancer 94: 624–632 11857293 10.1002/cncr.10226

[bib28] Strobel P, Bauer A, Puppe B, Kraushaar T, Krein A, Toyka K, Gold R, Semik M, Kiefer R, Nix W, Schalke B, Muller-Hermelink HK, Marx A (2004) Tumor recurrence and survival in patients treated for thymomas and thymic squamous cell carcinomas: a retrospective analysis. J Clin Oncol 22: 1501–1509 15084623 10.1200/JCO.2004.10.113

[bib29] Thomas CR, Wright CD, Loehrer PJ (1999) Thymoma: state of the art. J Clin Oncol 17: 2280–2289 10561285 10.1200/JCO.1999.17.7.2280

[bib30] Tobinai K, Kohno A, Shimada Y, Watanabe T, Tamura T, Takeyama K, Narabayashi M, Fukutomi T, Kondo H, Shimoyama M, Suemasu K (1993) Toxicity grading criteria of the Japan Clinical Oncology Group. The Clinical Trial Review Committee of the Japan Clinical Oncology Group. Jpn J Clin Oncol 23: 250–257 8411739

[bib31] Travis WB, Brambilla E, Muller-Hermelinck HK, Harris CC (2004) Pathology and Genetics of Tumours of the Lung, Pleura, Thymus and Heart. IARC Press: Lyon

[bib32] Urgesi A, Monetti U, Rossi G, Ricardi U, Casadio C (1990) Role of radiation therapy in locally advanced thymoma. Radiother Oncol 19: 273–280 2126388 10.1016/0167-8140(90)90154-o

[bib33] Yokoi K, Matsuguma H, Nakahara R, Kondo T, Kamiyama Y, Mori K, Miyazawa N (2007) Multidisciplinary treatment for advanced invasive thymoma with cisplatin, doxorubicin, and methylprednisolone. J Thorac Oncol 2: 73–78 17410014 10.1097/JTO.0b013e31802bafc8

